# Understanding professional development challenges of Chinese public health professionals: association and prediction analyses with data validity screening

**DOI:** 10.3389/fpubh.2023.1250606

**Published:** 2023-08-31

**Authors:** Yingchen Wang, Xiangran Kong, Fang Li, Hongyan Zhao

**Affiliations:** ^1^Shandong Youth University of Political Science, Jinan, China; ^2^Central Hospital Affiliated to Shandong First Medical University, Jinan, China

**Keywords:** professional development challenge, Chinese public health professional, chi-square test of association, logistic regression, Rasch model, careless responses, survey data quality

## Abstract

**Background:**

Little is known about the public health professionals engaged in educating and training new or future researchers in public health. Research in this direction identifies their issues, concerns, challenges, and needs. This study focused on the professional development challenges of Chinese public health professionals.

**Methods:**

Snowball sampling was utilized. A total of 265 public health professionals participated. An instrument of 6 dimensions (burnout, sleep issue, mood issue, friends’ support, exercise, and challenges) was developed, revised, and administered online. Two different approaches, the conventional and data screening approaches, were applied. The former started with item quality analyses, whereas the latter began with data quality checks. The chi-square tests of associations and logistic regressions were performed on both approaches.

**Results and discussion:**

19.25% of the participants were detected and deleted as careless respondents. Using both approaches, six professional development challenges except one (“Multidisciplinary learning”) were significantly associated with various demographic features. The two approaches produced different models though they converged sometimes. The latent variables of exercise predicted professional development challenges more frequently than other latent variables. Regarding correct classification rates, results from the data screening approach were comparable to those from the conventional approach.

**Conclusion:**

The latent variables of exercise, such as “Exercise effects,” “Expectations of exercise,” and “Belief in exercise,” might be understudied. More research is necessary for professional development challenges using exercise as a multidimensional construct. Based on the current study, screening and deleting careless responses in survey research is necessary.

## 1. Introduction

Public health professionals prevent and cure diseases and promote the public’s well-being. Their research refreshes our understanding of health-related issues, discovers more scientific ways to treat and prevent diseases, and builds various frameworks to understand our health. Their research impacts policy, our longevity, and quality of life. However, more is needed to know about the professional development of public health professionals engaged in training and educating new and future researchers (hereafter referred to as public health professionals). Research in this line is vital to understanding their professional issues, needs, and well-being, promoting their professional growth, and maximizing general welfare.

Chinese public health professionals have contributed tremendously to the Chinese people’s well-being. According to Chen et al. (1), life expectancy in mainland China increased by 9.44 years from 1990 to 76.3 years in 2016, and infant mortality dropped remarkably from 1990 to 2002. Behind these simple statistics are the efforts of numerous public health workforce at different levels in various disciplines. Their research plays a vital role in preventing diseases, prolonging the life of the Chinese people, and reducing mortality and morbidity. It is of practical significance that we understand the professional development of Chinese public health professionals for their well-being and the public benefits. However, there is a dearth of research on this topic.

The current research aimed to understand the professional development challenges of public health professionals in mainland China. For this purpose, snowball sampling was utilized to recruit public health professionals from hospitals and universities. A total of 265 filled out the online survey. First, tests of associations were conducted on their demographic variables and self-reported challenges in professional development. Next, logistic regressions were performed to examine the significant predictors for the self-reported challenges in professional development. More importantly, the current research applied data screening techniques in survey research to identify careless responses and remove these invalid data for high-quality research. The conventional approach and the data screening approach were used simultaneously.

## 2. Literature review

The current research incorporated the professional development framework with burnout, a common occupational phenomenon, to investigate the challenges of Chinese public health professionals. Furthermore, screening for data quality in survey research was introduced into the study. This section elaborated on professional development and the relationship between burnout (including its related consequences) and professional development.

### 2.1. Professional development

#### 2.2.1. Definition and importance

Professional development (PD) is a well-designed, systematic process to assist people in learning, retaining, and applying knowledge and skills related to their jobs (2). Professional development aims to teach new skills, knowledge, and strategies to bring positive career outcomes. It ensures quality improvement in public health care delivery and maintenance of public health (2). It can happen in different formats and levels. As such, professional development plays a vital role in enhancing the competencies of public health professionals in various disciplines. Thus, the PD program is popular in the public health sector. For instance, American Public Health Association established the Center for Public Health Practice and Professional Development.^1^

#### 2.2.2. Impacts of professional development

The literature consistently documents the positive impacts of PD. It was related to career satisfaction and quality patient care among nurses (3). Teachers who experienced PD programs had higher job satisfaction (4). Higher job satisfaction was associated with higher productivity and a lower turnover rate. Rouleau et al. (5) synthesized that the nurses participating in a PD program reported the most outcomes in learning and that participants perceived positive outcomes for older patients. Forsetlund et al. (6) reported that PD meetings improved the professional practices of health personnel and patient outcomes. To conclude, PD is essential in assisting health professionals to advance their skills and expertise and improve their professional practices.

#### 2.2.3. Exercise, friend influences, and professional development

Learning is central to PD. In education, theory and empirical evidence support the pivotal role of self-efficacy, which is an individual’s belief in their ability to succeed (7). People with a higher sense of self-efficacy can recover from setbacks more quickly, are more likely to take on challenges, and persist in difficulty. One of the sources of self-efficacy is vicarious influences, alternatively known as the role model from friends. The role model plays an important in the development of self-efficacy (8). Research demonstrated that exercise behaviors had strong predictive power on self-efficacy (9). Exercises improve psychological well-being, thus elevating the self-efficacy level of individuals (10, 11). Exercise positively affects the body, mind, and memory, improving learning (12). The national research report confirmed that exercise and support from friends could decrease burnout, facilitating professional development (13).

#### 2.2.4. Professional development challenge and the role of challenge

“Challenge” refers to something difficult or a task stimulating participants to reach some learning objectives or meet some criteria (14). In chess, sports, and leisure activities, researchers reported a positive association between challenges and enjoyment and between challenges and intrinsic goal orientations (15). In higher education, the challenge-based learning approach has been proven useful in fostering learning and learning outcomes (16–18). In some workplace training, challenge-based teaching was superior to lecture-based teaching, with more participant interaction and better learning outcomes (19). A challenge can stimulate an individual’s motivation to engage and pave the way to accumulate more expertise and knowledge, preparing an individual for the future. Therefore, it is meaningful to understand the self-perceived challenges of public health professionals.

### 2.2. Burnout and professional development

#### 2.2.1. Burnout definition and prevalence

Burnout is work-related stress characterized by physical and mental exhaustion, cynicism, and reduced professional efficacy (13). Burnout is common among public health sectors. The national report summarized that the burnout prevalence rate was 35% and 54% for American nurses and physicians and 45% and 60% for medical students and residents (13). In a Chinese cross-sectional study of mental health professionals, 38.1% of the participants suffered from burnout (20). Stone et al. (21) reported 66% of burnout in a sample of about 200 frontline public healthcare workers during the pandemic. Its prevalence differs by gender, cultural background, and age (13, 22).

#### 2.2.2. Burnout as a barrier to professional development

Research has consistently documented that occupational burnout was associated with sleep issues, suicide risks, higher rates of alcohol use, higher risks of depression, and sub-optimal professional outcomes among the healthcare workforce (13). A meta-analysis showed that, among health workers, the pooled prevalences of anxiety, depression, and sleep problems were 300%, 311%, and 440% during the pandemic (23).

Individuals with burnout were likely to decrease their professional engagement. Burnout was significantly associated with self-reported medical errors (24). Physicians with burnout syndrome were likely to have decreased motivation, a reduced sense of control over their practices, and experience suboptimal professional behaviors with patients and colleagues (25). Sleep disorders were also positively associated with medication errors and significantly impacted depression scores (26). Depressive physicians had a significantly higher risk of making medical errors (27). Chronic burnout strengthens emotional exhaustion and undermines daily functioning (28). The consequences of burnout differed by gender and background (13, 22).

#### 2.2.3. Professional development to alleviate burnout

The national report suggested positive learning environments to promote PD and reduce burnout (13). The research found that attitudes toward PD had a positive relationship with professional efficacy, a negative association with cynicism, and that attitudes differed by gender and participant experience (29). Professional development significantly predicts the burnout of healthcare workers (30). Attitudes toward professional development were positively related to the dimensions of burnout (31). Existing research supports the effectiveness of PD programs in reducing burnout. For example, the PD intervention decreased burnout and increased job satisfaction (32). Another PD program significantly reduced the perceived stress and burnout of the participating professionals (33).

### 2.3. Careless responses in survey research

Educational, sociology, psychology, and public health researchers utilize surveys as an essential vehicle for collecting and analyzing data. Survey data quality depends on how honestly the participants follow the instructions and complete the survey. The recent two decades have witnessed careless response (CR) research growth.

#### 2.3.1. Definition and prevalence of careless responses

CR is present when participants are not motivated to provide accurate or correct choices. CR can be either random or non-random. Random CR is a response pattern in which participants randomly fill out the survey (34). Non-random CR is the behavior of choosing the same options in a highly consistent manner (34). CR prevails in survey research. Hong et al. (35) reported that the CR rates ranged from 20% to 50% in the reviewed articles. Their research had about 33% of CR. Ward et al. (36) reported CR as a common source of bias in online surveys. With technological innovations, online surveys are becoming popular. It is vital to examine the data quality of our online survey.

#### 2.3.2. Consequences of careless responses

When CR is present, it compromises data quality and distorts statistical results and research conclusions. Huang et al. (37) found that CR increased the correlation among variables and inflated the Type I error rate. Goldammer et al. (38) reported that CR increased item variance and pulled item means toward midpoints. Some research reported that CR obscured the significance of treatment (39). Kam (40) detected that CR distorted factor loadings and threatened construct validity. CR produced bias in item parameter estimates and spuriously decreased the standard error estimates (41). Scholars recommend removing CRs to ensure high-quality data (38, 42, 43).

#### 2.3.3. Growing practices

Some researchers have used data screening techniques to improve data quality. Osborne and Blanchard (39) removed the CR. The intervention in their study became significant. Kam (40) reported that the sample of careful respondents showed more substantial evidence of data validity than the sample with careless responses. For some research, removing CR increased the credibility of the findings (44). However, data screening and removal have yet to catch enough attention (40, 45).

Following this trend, the current study adopted two different approaches to data analysis. First was the conventional method. We conducted item quality analyses before other statistical analyses. Second was the data screening approach with data quality checks and item quality research before performing any analyses. The specific questions were as follows.

### 2.4. Research questions:

1. What demographic characteristics were associated with Chinese public health professionals’ professional development challenges? The chi-square test of association was used to answer the question.
2. What predicted the self-perceived challenges of professional development? The logistic regression was applied to examine the predictive power of the predictors.

The results from the two different approaches were discussed in section 4. Section 3 presents the details of the research methodology.

## 3. Materials and methods

### 3.1. Instrument and sample

Institutional research board (IRB) approval was obtained from the university. At the very beginning of the survey, the participants were informed of the aims and uses of the data and the researchers’ promise of confidentiality. The directions at the beginning of the study emphasized that only the public health professionals engaged in educating, administering, and training new and future researchers were eligible to participate and that each individual needed to fill out the survey once. The initial survey had 70 items. Some participants in the pilot test suggested reducing the number of items to recruit more people. Thus, the ten items on general self-efficacy were removed.

The administered version had 60 items with six dimensions—burnout, sleep issues, mood issues, professional development challenges, support from friends, and physical exercise. The six dimensions were developed based on the literature related to PD and burnout. The research pinpoints sleep and mood issues as expected outcomes of burnout. It confirms that support from friends and exercises are instrumental for self-efficacy development. Demographic items were at the end of the survey. The burnout items were based on the Chinese version of the Maslach Burnout Inventory General Survey (MBI-GS) (46) and Likert-scaled, addressing emotional exhaustion (5 items), cynicism (4 items), and professional efficacy (9 items). The options ranged from 1 (“Very few, several times a year”) to 6 (“Every day”). Questions for sleep, mood, and professional development challenges were dichotomous (“No”/“Yes”). Support from friends and exercise comprised six items and 16 items, respectively. Each item for “Friends” had a 4-point scale, including “Very few,” “Sometimes,” “Often,” and “Always.” Exercise items were further conceptualized to include the exercise effects (6 items), the expectations of exercise (5 items), and belief in exercise (5 items). Each item had five options, including “Strongly Disagree” to “Strongly Agree”.

The survey was administered on Wenjuanxing (a Chinese survey website). Snowball sampling was used to recruit participants from hospitals and universities in different regions. The co-authors distributed the survey among their co-workers and friends, who forwarded it to other professionals who met the inclusion criteria. The final sample size was 265.

### 3.2. Variables

The dependent variables were PD challenges in various aspects. They included the challenges of peer cooperation, multidisciplinary cooperation, physical and mental exhaustion, learning new knowledge and skills in one’s major, multidisciplinary learning, improving individual jobs, professional communication with peers, and helping students’ academic growth. The rest variables were independent.

MBI-GS had 15 items. For emotional exhaustion, some items were “My job makes me exhausted” and “I feel burnout at the end of the day.” Cynicism included four items, such as “I doubt the significance of my job.” There were six items for professional efficacy, such as “I have done much valuable work”.

Sleep and mood items investigated if the participants had any disorders in the related area(s). The sleep questions included insomnia, easiness of waking up, drowsiness, fatigue after waking up, and early wakening. Mood questions examined the tendency to become irritable, angry, lose emotional control, and unhappy or low spirit over 15 days.

Items for support from friends asked participants to answer how many friends they had, how frequently they met, how involved their communications were over work-related issues, and how frequent conversations were over other topics such as personal issues and their feelings.

As mentioned previously, there were three different sub-dimensions with exercise. The exercise effects dealt with the frequency of exercise, the time length of each time they exercised, and the feelings after exercise. Two of the items were negatively worded. Thus, reverse coding was done after data collection. The expectations of exercise focused on participants’ subjective expectations when they exercised. For example, “I expect myself to burn a certain amount of calories each time I excise.” Each question in the exercise belief dimension emphasized belief. For example, “I believe that exercise can strengthen my stamina”.

Demographic variables included (1) gender, (2) job types (doctoral or master advisor, not an academic advisor but teaches doctoral or master courses, lab manager, administrative personnel, director or deputy chief physician/nurse, intermediate-level physicians/nurse, elementary-level physician or nurse), (3) institution (hospital versus university), (4) education (doctoral, master, bachelor, two-year college degree, below two-year college degree), (5) marital status (married, divorced, or single), and (6) the number of years in the profession. Due to the low frequency in the “Divorced” category, it was combined with “Single,” forming the “Unmarried” category. For the same reason, “Below two-year college degree” was combined with “Two-year college degree”.

### 3.3. Statistical analyses

**Figure 1:**
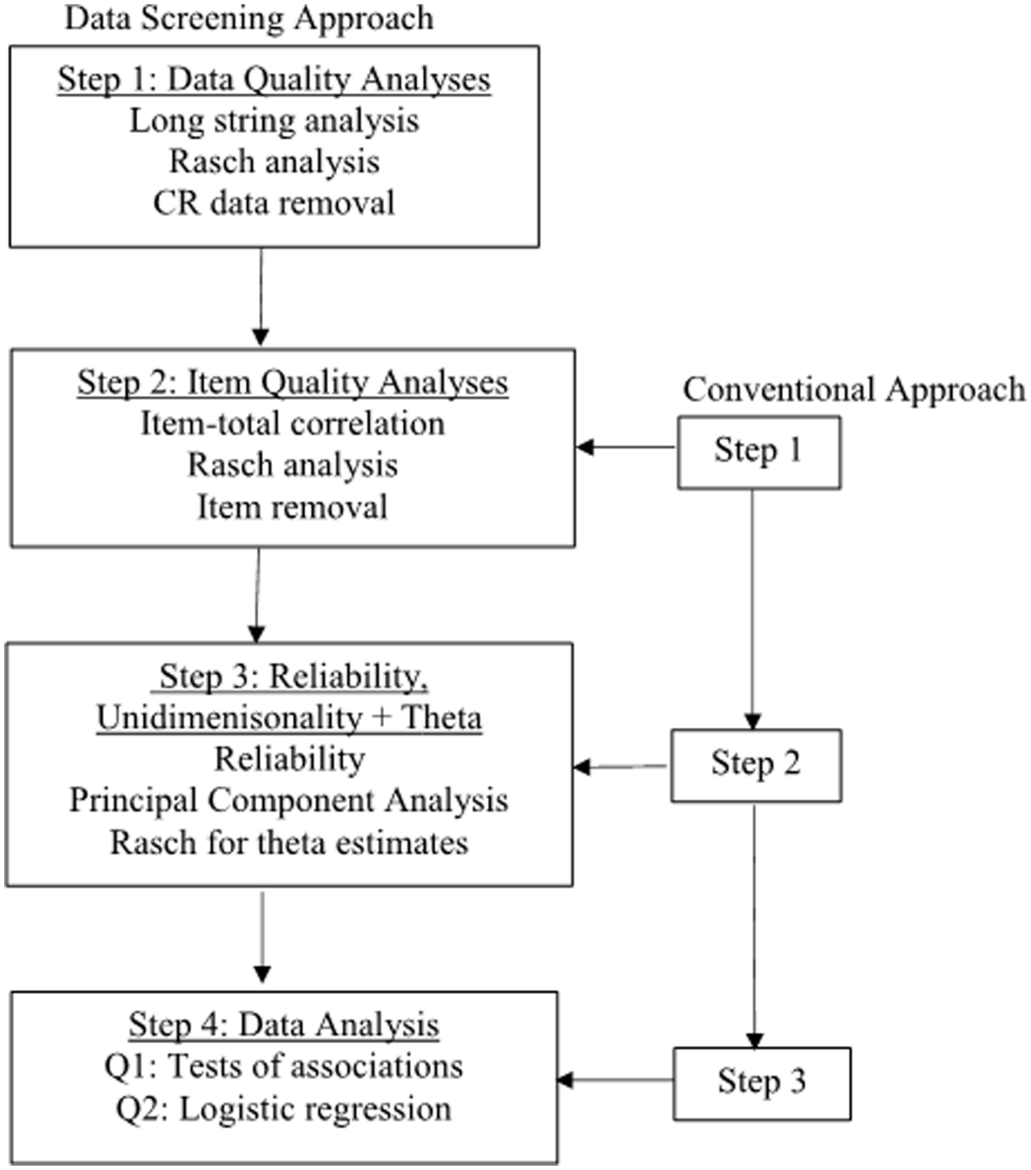
Flow chart for analytical procedures. Each step in the conventional approach is one step behind the data screening approach.

Figure 1 presents the analytical procedures for the two approaches. The data screening approach started with the data quality check, i.e., CR analyses. The conventional approach skipped CR analyses, began with the item quality analyses, and did the analyses on the data after poor-quality items were removed (hereafter referred to as the conventional dataset). The following paragraphs elaborate on the details of the data screening approach.

#### 3.3.1. Step 1 for data quality check—CR analyses

In this step, we conducted CR analyses to detect and remove CR. Multiple techniques exist for detecting careless responses. Curran (34) and Hong et al. (35) proposed using at least two techniques. Thus, we selected long string and Rasch outlier analyses. The former detects non-random CR, and the latter random CR. Additionally, the stopping criterion exists for the Rasch analysis (see 3.3.5). For the screening approach, the data quality analyses were performed on MBI-GS for several reasons. First, careless responses occur in Likert-scale surveys. There were six different dimensions in our instrument. The number of options varied with each dimension, and some were dichotomous. Conducting the long string analysis on the total items was not feasible. Second, our instrument was brief. The participants’ attitudes should remain unchanged from the beginning to the end. Details for each screening technique were as follows.

**Figure 2:**
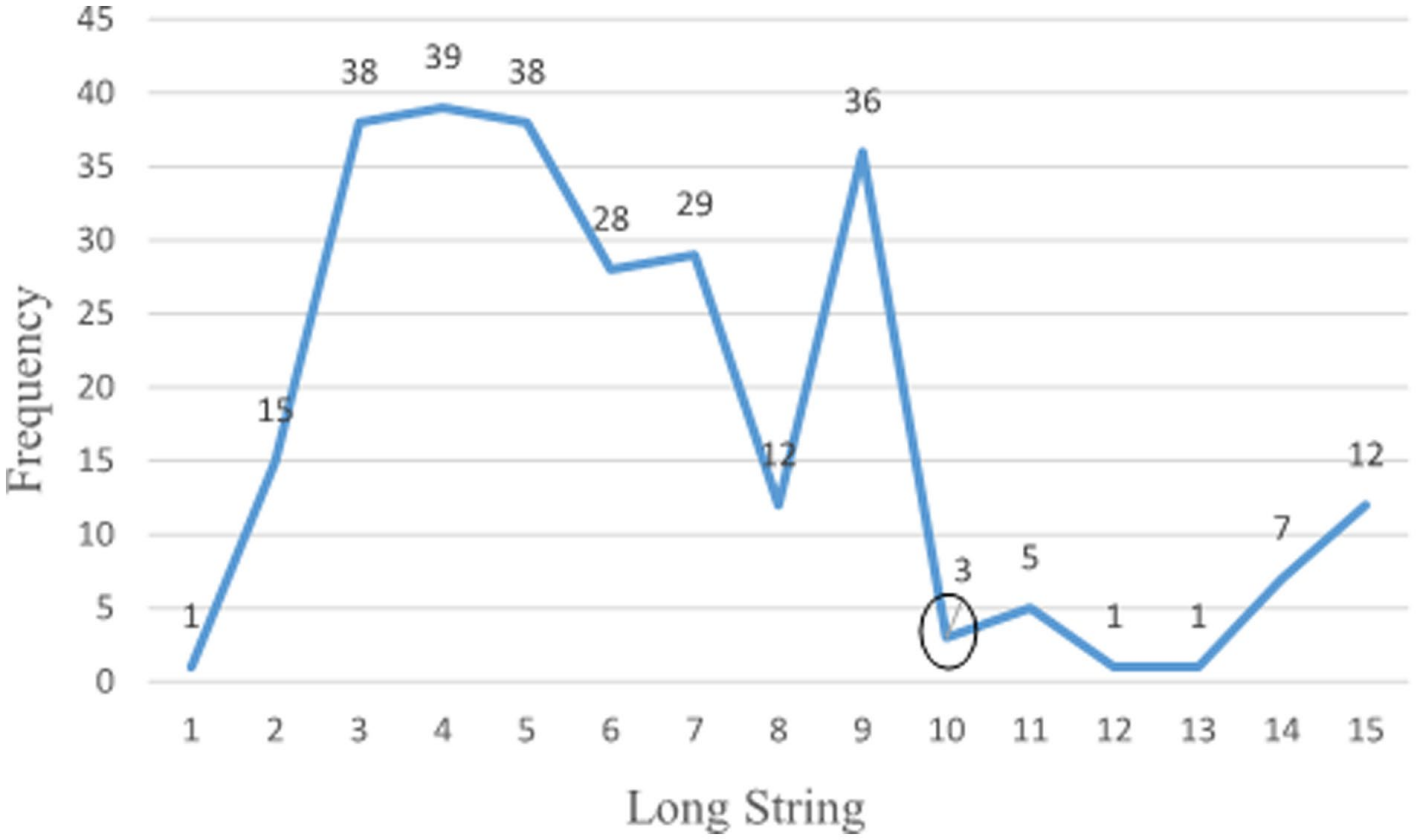
Long string frequency.

1. Longstring for non-random CR. Non-random CR is the overly consistent response pattern (34, 35, 47). Respondents fail to give enough attention to the survey content. Longstring analysis helps to identify some severely careless responses (28). A long string of consistent options for at least half the total scale length can be considered careless responses (28). Johnson (47) and Niessen et al. (43) chose the maximum long string as cutoff values. We determined that when respondents chose ten same consecutive options out of the 15 MBI-GS items, they were careless respondents (Figure 2 in 4.1.1).
2. Rasch outlier analysis for random CR. Random careless responses are random selections, like flipping a coin. Osborne and Blanchard (39) proved that the Rasch outfit index was as sensitive to random responses as another statistic. To perform Rasch analysis, several steps were necessary.

First, the number of response options for some items was combined, reducing the categories from six to five to ensure that each response category had enough respondents. According to Linacre’s (48) guideline, there should be a minimum of 10 respondents per category for the Likert-scaled items. This size guarantees that the precision of item and person parameter estimation falls within a ± logit confidence interval.

Second, random CR analyses were performed using Rasch outfit fit statistics. Rasch fit statistics on MBI-GS were obtained using jMetrik (49). jMetrik is a free computer program for classical and modern psychological model analyses. Meyer and Hailey (50) verified that jMetrik and WINSTEPS yielded similar results with different sample sizes and items. For sample size <300, standardized outfit statistics > |3.0| indicate outliers (personal communications with M. Linacre, Ph.D., Research Director, mike@winsteps.com, on March 09, 2023) and were applied to detect random CR. The participants detected as careless respondents using longstring or outlier analyses were removed from research in the screening approach.

#### 3.3.2. Step 2 for item quality check—item analyses

Two different statistics were utilized. First was the item-total correlation. A negative item-total correlation means that the item measures another trait from the remaining items and should be removed. The results revealed that all item-total correlations were positive. Second, Rasch infit and outfit statistics were obtained after merging adjacent categories of some items to retain enough respondents. After CR was removed, the sample size decreased. Thus, further combinations of adjacent categories for some items were performed. jMetrik was re-run on the screened data to get item fit statistics. Items with standardized outfit statistics > |3.0| were removed from the analyses. Due to the multi-dimensionality nature of the dataset, item-total correlation and Rasch model were run separately on each dimension.

#### 3.3.3. Step 3 for reliability, unidimensionality, and theta (i.e., estimated ability)

Two public health experts discussed the instrument’s content validity, covering each item’s validity, dimension validity, and other potential issues. After removing misfit items or CR, Cronbach alpha for each dimension was calculated for both data types. These dimensions included all burnout items, sleep, mood, friend, and all exercise items. Principal component analysis (PCA) was performed on each dimension to investigate the unidimensionality of related dimensions. The fundamental assumption of the Rasch model is that the data is unidimensional.

When unidimensionality was confirmed, jMetrik was performed on each dimension or sub-dimension to obtain each individual’s estimated theta for prediction analysis. These thetas included sleep issues, mood issues, support of friends, the three sub-dimensions of burnout, and the three sub-dimensions of exercise. The estimates from the Rasch model have several advantages over the observed data. First, the observed data were ordinal and might run into small frequencies with some items; thus, the results might be biased with low power. In contrast, the Rasch model produces continuous estimated ability, thus avoiding the small frequency issue with ordinal data. Second, the Rasch models are well-known for their robustness in the case of the small sample (51, 52).

#### 3.3.4. Step 4 for association and logistic regression analyses

Chi-square tests examined the associations between the challenges and the demographic variables. For question 2, four sets of variables were hierarchically entered into the model, and the forward stepwise selection method was utilized to investigate which variable(s) in each set contributed significantly to predicting the perceived challenges of Chinese public health professionals. The entry level in our study was set at 0.10, and the removal level at 0.15. The first set was demographic features. The second set was sleep, mood, and support from friends. The third set was the exercise effects, the expectations for doing exercise, and the belief in the benefits of exercise. The last set was the three subdimensions of burnout. The 2nd to fourth set variables were the estimated thetas from the Rasch model. Tests of associations and logistic regression analyses were performed using SPSS 22.0.

#### 3.3.5. Stopping and evaluation criteria for analyses

Linacre (53, 54) provided a guideline for removing items. His suggestions are to start deleting the item with the worst fit. The next step is to rerun the analysis and cross-plot the thetas from this step with those in the previous step. If the scatter plot reveals no noticeable changes, we should accept the items in the last step. If the plot shows noticeable changes, we should remove the item and perform Rasch analysis again. In the coming phase, if there are any misfit items, remove the worst, and do the persons’ estimates. Then, we cross-plot again and repeat what we have done in the preceding steps. When the differences in the person’s estimates between the current and previous stages are small, we can stop. Linacre (53, 54) suggested the same guideline for removing misfit persons.

For dimensionality analysis in 2.3.3, two indexes were used simultaneously to evaluate the dimensionality of the PCA results. The first was the number of components exacted. The second index was utilized when the number of extracted components exceeded 1.0. It was the ratio of the first-to-second eigenvalue. A ratio > 3.0 suggests multi-dimensionality.

For association analyses, two significance levels, 0.05 and 0.10, were chosen to compare the analytical results from the conventional approach against those from the data screening approach. The purpose was to highlight which approach yielded more significant results.

For logistic regression, only the indexes for the final selected model in each logistic regression were reported. Specific evaluation criteria included:

1. Which predictors were significant? What were the odds ratio (OR) and the confidence interval (CI) for the significant preditors? When CI for the odds ratio includes 1.0, it suggests non-significance. When CI excludes 1.0, it indicates significance.
2. What were the sizes of Nagelkerke R-square (hereafter referred to as the R-square) and Hosmer and Lemeshow goodness-of-fit test (hereafter referred to as GOF test) results? The former is about the amount of variability the model explains, and the nonsignificant value of the latter suggests model fit.
3. Last, we examined the percentage of correctly classified participants for each logistic regression. If the two approaches produce comparable results, it indicates that the data screening approach removed invalid data.

## 4. Results

### 4.1. Preliminary results and descriptive statistics

#### 4.1.1. Data screening results

22 (8.30%) participants were identified as random respondents or outliers, using the standardized outfit value > |3.0|. Figure 2 presents the longstring frequency. 29 (10.94%) respondents were flagged as non-random respondents. The two screening techniques excluded 51 (19.25%) participants. Table 1 compares demographics for the conventional and screened datasets. After data screening and removal, the sample size decreased from 265 to 214. For the original data, the frequency ranged from 178 to 14. For the screened dataset, the frequency ranged from 154 to 12.

**Table 1:** Demographic characteristics.

Variable	Level	Conventional data (*N*=265)		Screened data (*N*=214)	
		Frequency	Percentage	Frequency	Percentage
Sex	Male	93	35.1%	65	30.4%
	Female	172	64.9%	149	69.6%
Institution	Hospital	133	50.2%	116	54.2%
	University	132	49.8%	98	45.8%
Job type	Doctoral/master advisor	36	13.6%	29	13.6%
	Teaches doctoral or master's courses	24	9.1%	21	9.8%
	Laboratory manager	14	5.3%	12	5.6%
	Administrative personnel	19	7.2%	15	7.0%
	Director or deputy chief physician/nurse	60	22.6%	52	24.3%
	Intermediate-level physicians or nurses	55	20.8%	41	19.2%
	Elementary-level physicians or nurses	57	21.5%	44	20.6%
Education	At or below 2-year college degree	59	22.3%	37	17.3%
	Bachelor degree	65	24.5%	57	26.6%
	Master degree	78	29.4%	71	33.2%
	Doctoral degree	63	23.8%	49	22.9%
Marital status	Unmarried	87	32.8%	60	28.0%
	Married	178	67.2%	154	72.0%
Years of work	<1 Year	44	16.6%	27	12.6%
	1–3 Years	27	10.2%	23	10.7%
	3–5 Years	28	10.6%	20	9.3%
	5–7 Years	16	6.0%	13	6.1%
	7–10 Years	24	9.1%	19	8.9%
	>10 Years	126	47.5%	112	52.3%

#### 4.1.2. Item quality analytical results

The Rasch model was run on the conventional dataset for item quality purposes. According to the stopping criteria (3.3.5 Stopping and Evaluation Criteria for Analyses), an item was removed if the scatterplot of thetas demonstrated any noticeable changes. The four items were removed: (1) “It is easy for me to become angry.” (2) “It is challenging to deal with physical and mental exhaustion.”; (3) “I can communicate with my friends about nonwork-related problems (such as family, spouse, children, etc.).”; and (4) “Each time I exercise, I expect myself to burn a certain amount of calories”.

After cleaning the invalid data, jMetrik was rerun on the cleaned dataset. The five items were removed. The first three were the same as the three in the conventional dataset. One challenge item (“Across-department cooperation is a challenge”) was deleted. One exercise item (“How long do you exercise each time”) was deleted.

#### 4.1.3. Reliability and unidimensionality

Reliability and validity are essential indexes of data quality. Except for the reliability of mood (0.576) with the screening approach, all other reliability indexes were acceptable, ranging from 0.615 to 0.894. With burnout as one dimension, the reliability for the conventional dataset was 0.831 and 0.726 for the screened dataset. The reliability index for sleep was 0.67 for the conventional dataset and 0.615 for the screened dataset. The reliability index for mood was 0.649 for the conventional dataset. For the friends, the index changed from 0.712 for the conventional data to 0.657 for the screened data. Last, the reliability was 0.894 and 0.893 for all exercise items. In general, the screened data exhibited lower reliability than the unscreened data. For the lowest reliability, only three items were available for Cronbach’s alpha. Increasing the number of items will increase the reliability.

Two subject experts examined the survey items and confirmed the content validity of each item and dimension. In addition, we conducted principal component analyses (PCA) for the original and the screened datasets. The PCA produced only one component for “Emotional exhaustion,” “Cynicism,” “Professional efficacy,” “Mood,” “Support from friends,” “Expectations of exercise,” and “Belief in exercise.” PCA on sleep and exercise effects produced two extracted principal components for both data types. However, the ratios of the first-to-second eigenvalue were all <3.0. Thus, the unidimensionality assumption was met.

#### 4.1.4. Descriptive statistics for the estimated thetas

The descriptive statistics in Table 2 present the means and standard deviations of the thetas from the Rasch model. Comparing the two data types, the screened data exhibited higher means of “Emotional exhaustion” and “Cynicism” and a lower mean of “Professional efficacy,” indicating more problems with burnout. The screening lowered the means for sleep disorders and mood issues and a slightly higher mean for support from friends. Higher values suggest more sleep and mood problems and more support from friends. Lower means suggest fewer sleep and mood issues and less help from friends. The screening lowered the means of the three different dimensions of exercise. Lower values in these three dimensions suggest less impact of exercise on individual life.

**Table 2:** Descriptive statistics for estimated thetas.

Statistics estimates	Conventional data (*N*=265)				Screened data (*N*=214)			
	Mean	SD	Mini	Maxi	Mean	SD	Mini	Maxi
Emotional exhaustion	−2.702	3.679	−7.968	8.309	−1.649	2.975	−6.443	5.344
Cynicism	−3.101	3.221	−6.372	5.049	−2.112	2.353	−4.588	3.953
Professional efficacy	1.120	3.401	−6.457	6.573	0.683	2.840	−5.766	5.781
Sleep	−0.661	1.752	−2.900	2.911	−0.753	1.644	−2.885	2.901
Mood	−0.926	2.001	−2.785	2.950	−1.049	1.898	−2.815	2.986
Friend		1.453	−4.016	3.841	−0.670	1.311	−4.023	2.621
Effects of exercise	0.198	1.288	−4.430	4.621	0.171	1.347	−4.517	4.602
Expectations of exercise	−0.481	2.393	−4.165	5.262	−0.574	2.800	−4.587	5.885
Belief of exercise	2.471	5.165	−9.412	9.186	1.629	4.943	−10.026	7.976

SD, standard deviation.

### 4.2. Association tests

Table 3 displays the *p*-values for the tests of associations for both data types. “Improving my job” was significantly associated with job type, education level, and years of work for the conventional dataset. It was significantly associated with job type and years of work for the screened dataset. “Professional communication with coworkers” was significantly related to marital status and years of work for the conventional dataset. It was significantly associated with education level, marital status, and years of work for the screened dataset. These two challenges were associated with more demographic variables. Next came “Peer cooperation.” It was significantly associated with gender and years of work in the two analytical approaches. “Learning new knowledge or skills in my major” was significantly related to institutions for both data types. Last, “Helping students’ academic growth” was significantly associated with the institution variable.

**Table 3:** *P*-values for tests of associations.

Variables	Challenge 1	Challenge 4	Challenge 5	Challenge 6	Challenge 7	Challenge 8
Conventional data						
Sex	**0.012**					
Institution		**0.042**				**0.002**
Job type				**0.036**		
Education				**0.019**		
Marriage					**0.023**	
Years of work	**0.026**			*0.097*	*0.058*	
Screened data						
Sex	0.053^**^					
Institution		0.069^**^				0.001^*^
Job type				0.068^**^		
Education				***	0.064^*^	
Marriage					0.001^*^	
Years of work	0.022^*^			0.024^*^	0.018^*^	

**Bolded** numbers indicated significance at 0.05 level and *italicized* numbers at 0.10 level. ^*^ indicate that either the *p*-values became smaller or became significant at 0.05 or 0.10 level with the screened data. ^**^ indicate the *p*-values became bigger with the screened data. ^***^ indicate the test became insignificant with the screened data. Challenge 1: peer cooperation. Challenge 4: learning new knowledge or skills in my major. Challenge 5: multidisciplinary learning. Challenge 6: improving my own job. Challenge 7: professional communication with peers. Challenge 8: helping students’ academic growth.

### 4.3. Logistic regression

Based on the item analytical results, two challenge items were removed from the screened dataset and one from the conventional dataset. Logistic regression was run on the remaining six challenge items for comparison purposes. SPSS modeled “Yes” as the event. All the demographic variables were dummy coded with the last category as the reference group. In this section, OR was interpreted, holding all other variables in the model constant.

#### 4.3.1. Prediction for peer cooperation

The results are presented in Table 4. For the *conventional dataset*, compared with those working more than ten years, those with 1–3 years had 4.781 times the odds of selecting peer cooperation as a challenge (CI: 1.882, 12.141). For each unit increase in “Sleep,” we expected about a 39% increase in selecting “Yes” for peer cooperation. CI for this odds ratio excluded 1.0. For each unit increase in “Expectations of exercise,” there would be about a 12% increase in endorsing peer cooperation as a challenge. Its CI included 1.0. For every unit increase in support from friends, we expected about an 82% decrease in selecting this challenge (CI: 0.670, 0.996). The R-square was 0.195, and the GOF was 0.667.

**Table 4:** Logistic regression for peer cooperation.

Block	Variable	*B*	S.E.	Wald	OR	95% CI for OR	
						Lower	Upper
Conventional data							
Final model	Sex (1)	*0.570*	0.299	3.629	1.769	0.984	3.181
	Years of work			**13.222**			
	Years of work (1)	0.126	0.413	0.093	1.135	0.505	2.551
	Years of work (2)	**1.565**	0.476	10.826	4.781	1.882	12.141
	Years of work (3)	0.246	0.477	0.266	1.279	0.502	3.258
	Years of work (4)	−0.307	0.652	0.223	0.735	0.205	2.637
	Years of work (5)	*0.813*	0.495	2.705	2.256	0.856	5.946
	Sleep	**0.331**	0.083	15.764	1.392	1.183	1.640
	Friend	**−0.202**	0.101	4.010	0.817	0.670	0.996
	Expectations of exercise	*0.114*	0.061	3.498	1.121	0.995	1.264
	Intercept	**−1.031**	0.242	18.201	0.357		
Screened data							
Final model	Years of work			**13.453**			
	Years of work (1)	0.227	0.463	0.240	1.255	0.506	3.112
	Years of work (2)	**1.625**	0.507	10.275	5.079	1.880	13.719
	Years of work (3)	−0.011	0.539	0.000	0.989	0.344	2.845
	Years of work (4)	−0.714	0.808	0.781	0.490	0.101	2.385
	Years of work (5)	0.864	0.535	2.610	2.372	0.832	6.762
	Sleep	**0.298**	0.097	9.378	1.347	1.113	1.629
	Intercept	**−0.683**	0.213	10.297	0.505		

**Bolded** parameter estimates or Wald statistics indicated significance at 0.05 level and *italicized* numbers at 0.10 level.

For the *screened dataset*, years of work significantly predicted the challenge of peer cooperation. Compared with those working more than ten years, those with 1–3 years of experience had 5.079 times the odds of selecting “Yes” for peer cooperation. For every unit increase in “Sleep,” the odds of choosing “Yes” increased by 1.347 (CI: 1.113 and 1.629). The R-square was 0.140, and the GOF was 0.437.

#### 4.3.2. Prediction for learning new knowledge or skills in my major

For the *conventional dataset* (the top part of Table 5), three variables, institution, “Mood,” and “Professional efficacy,” were all non-significant with CI including 1.0. The bottom part of Table 5 shows the results for the *screened data.* For every unit increase in “Professional efficacy,” we expected about a 0.87% decrease in selecting this challenge (CI: 0.786 and 0.966). The R-square for the conventional dataset was 0.056, and the GOF was 0.39. The R-square for the screened data was 0.073, and the GOF was 0.400.

**Table 5:** Logistic regression for learning new knowledge/skills in my major.

Block	Variable	*B*	S.E.	Wald	OR	95% CI for OR	
						Lower	Upper
Conventional data							
Final Model	Institution (1)	−0.284	0.274	1.076	0.752	0.440	1.288
	Mood	*0.114*	0.064	3.161	1.121	0.988	1.271
	Professional efficacy	−0*.071*	0.041	2.929	0.932	0.859	1.010
	Intercept	**0.414**	0.188	4.862	1.513		
Screened data							
Final Model	Mood	0.111	0.077	2.039	1.117	0.960	1.300
	Professional efficacy	**−0.138**	0.053	6.805	0.871	0.786	0.966
	Intercept	*0.268*	0.163	2.705	1.307		

**Bolded** numbers indicated significance at 0.05 level and *italicized* numbers at 0.10 level.

#### 4.3.3. Prediction for multidisciplinary learning

Table 6 reveals the results of multidisciplinary learning. For the *conventional data*, “Sleep” and “Effects of exercise” were in the model with CI including 1.0. For every unit increase in “Exercise expectations,” there would be about a 19% increase in selecting “Yes” for this challenge (CI: 1.066 and 1.338). In the *screened data*, for every unit increase in “Expectations of exercise,” we expected an 11% increase in choosing “Yes” for this challenge (CI: 1.003 and 1.222). The R-square was 0.079, and the GOF was 0.489 for the conventional approach. These indexes were 0.026 and 0.433 for the screened dataset.

**Table 6:** Logistic regression for multidisciplinary learning.

Block	Variable	*B*	S.E.	Wald	OR	95% CI for OR	
						Lower	Upper
Conventional data							
Final model	Sleep	*0.136*	0.077	3.101	1.145	0.985	1.332
	Effects of exercise	−0*.194*	0.113	2.986	0.823	0.660	1.026
	Expectations of exercise	**0.178**	0.058	9.381	1.194	1.066	1.338
	Intercept	**0.363**	0.142	6.579	1.438		
Screened data							
Final model	Expectations of exercise	**0.102**	0.050	4.068	1.107	1.003	1.222
	Intercept	0.173	0.142	1.494	1.189		

**Bolded** numbers indicated significance at 0.05 level and *italicized* numbers at 0.10 level.

#### 4.3.4. Prediction for improving my job

For the *conventional data* (the top part of Table 7), intermediate-level physicians and nurses had 2.603 times the odds of choosing “Improving my job” as a challenge compared with those elementary positions. Compared with professionals with doctoral degrees, those with master’s degrees had 2.815 times the odds of considering “Improving my job” as a challenge. CI for both odds ratios excluded 1.0. For every unit increase in “Exercise effects,” the odds for the predicted event decreased by 0.697 (CI: 0.530, 0.916). “Sleep” and “Belief in exercise” were nonsignificant. The R-square for the final model was 0.158, and the GOF was 0.447 for the original data.

**Table 7:** Logistic regression for improving my job.

Block	Variable	*B*	S.E.	Wald	OR	95% CI for OR	
						Lower	Upper
Conventional data							
Final model	Job type			**13.577**			
	Job type (1)	0.346	0.497	0.484	1.413	0.534	3.744
	Job type (2)	*1.066*	0.570	3.500	2.905	0.951	8.877
	Job type (3)	−0.758	0.681	1.241	0.468	0.123	1.778
	Job type (4)	−0.579	0.602	0.923	0.561	0.172	1.826
	Job type (5)	**0.957**	0.434	4.867	2.603	1.113	6.090
	Job type (6)	0.205	0.423	0.234	1.227	0.535	2.813
	Education			**8.788**			
	Education (1)	0.077	0.438	0.031	1.080	0.458	2.548
	Education (2)	0.490	0.424	1.332	1.632	0.710	3.749
	Education (3)	**1.035**	0.396	6.842	2.815	1.296	6.113
	Sleep	0.100	0.080	1.582	1.105	0.946	1.292
	Effects of exercise	**−0.361**	0.139	6.713	0.697	0.530	0.916
	Belief of exercise	*0.055*	0.032	2.921	1.056	0.992	1.125
	Intercept	−0*.785*	0.456	2.968	0.456		
Screened data							
Final model	Years of work			**12.976**			
	Years of work (1)	**−0.971**	0.458	4.502	0.379	0.154	0.929
	Years of work (2)	−0.185	0.471	0.154	0.831	0.331	2.090
	Years of work (3)	**−1.141**	0.521	4.803	0.320	0.115	0.886
	Years of work (4)	**−2.054**	0.798	6.622	0.128	0.027	0.613
	Years of work (5)	−0.443	0.503	0.777	0.642	0.239	1.720
	Cynicism	**0.142**	0.064	4.887	1.152	1.016	1.307
	Intercept	**0.713**	0.254	7.849	2.040		

**Bolded** parameter estimates or Wald statistics indicated significance at 0.05 level and *italicized* numbers at 0.10 level.

For the *screened data*, compared with those having >10 years of experience, those with 5–7 years of experience has 0.128 times the odds of choosing this challenge (CI: 0.027, 0.613). Compared with those with >10 years of experience, the odds of selecting this challenge decreased by 0.379 for professionals with <1 year of experience (CI: 0.154 and 0.929). Compared with those with >10 years of experience, the odds of this event for those with 3–5 years of experience decreased by 0.320 (CI: 0.115 and 0.886). Every unit increase in “Cynicism” increased the odds of selecting this challenge by 1.152 (CI: 1.016 and 1.307). The R-square for the final model was 0.111, and the GOF was 0.183.

#### 4.3.5. Prediction for communication with peers

Table 8 displays the results of the challenge of professional communication with peers. For the *conventional data*, for every unit increase in “Expectations of exercise,” we expected about a 25% increase in selecting “Yes” for this challenge (CI: 1.103 and 1.419). For every unit increase in “Emotional exhaustion,” we expected about an 11% increase in selecting “Yes” for this challenge (CI: 1.013, 1.210). Other variables in the model were non-significant. The R-square was 0.165, and the GOF was 0.552.

**Table 8:** Logistic regression for professional communication with peers.

Block	Variable	*B*	S.E.	Wald	OR	95% CI for OR	
						Lower	Upper
Conventional data							
Final model	Marital status (1)	0.470	0.295	2.525	1.599	0.896	2.854
	Mood	0.029	0.079	0.138	1.030	0.883	1.201
	Friend	−0.166	0.103	2.609	0.847	0.693	1.036
	Effects of exercise	−0.191	0.135	1.992	0.826	0.634	1.077
	Expectations of exercise	**0.224**	0.064	12.082	1.251	1.103	1.419
	Emotional exhaustion	**0.102**	0.045	5.072	1.107	1.013	1.210
	Intercept	−0.254	0.223	1.290	0.776		
Screened data							
Final model	Marital status (1)	**0.844**	0.329	6.572	2.326	1.220	4.435
	Expectations of exercise	**0.123**	0.054	5.299	1.131	1.019	1.257
	Cynicism	**0.154**	0.064	5.692	1.166	1.028	1.323
	Constant	−0.274	0.230	1.419	0.761		

**Bolded** numbers indicated significance at 0.05 level and *italicized* numbers at 0.10 level.

For the *screened data*, compared with the married professionals, those without marriages had 2.326 times the odds of choosing this challenge (CI: 1.220, 4.435). For every unit increase in “Expectations of exercise,” the odds of choosing “Yes” for this challenge increased by 1.131 (CI: 1:019, 1.257). For every unit increase in “Cynicism,” the odds of choosing “Yes” for this challenge increased by 1.166 (CI: 1.028 and 1.323). The R-square was 0.137, and the GOF was 0.178.

#### 4.3.6. Prediction for helping students’ academic growth

For the *conventional data* (the top part of Table 9), compared with the university professionals, the hospital professionals had 0.322 times the odds of choosing this challenge (CI: 0.165 and 0.629). Compared with those in elementary positions, director or deputy chief professionals had 6.238 times the odds of selecting this event (CI: 2.383, 16.329). With the same reference group, those teaching doctoral or master courses had 4.553 times the odds of endorsing this challenge (CI: 1.421, 14.594). For every unit increase in “Effects of exercise,” there was about a 46% decrease in selecting “Helping students’ academic growth” as a challenge (CI: 0.322, 0.649). For every unit increase in “Expectations of exercise,” there was about a 29% increase in choosing “Yes” (CI: 1.112, 1.493). For every unit increase in “Belief of exercise,” there was about an 11% increase in choosing “Yes” for this question (CI: 1.029, 1.196). The R-square was 0.30, and the GOF was 0.962.

**Table 9:** Logistic regression for helping student academic growth.

Block	Variable	*B*	S.E.	Wald	OR	95% CI for OR	
						Lower	Upper
Conventional data							
Final model	Institution (1)	**−1.132**	0.341	11.035	0.322	0.165	0.629
	Job type			**18.933**			
	Job type (1)	0.728	0.510	2.038	2.071	0.762	5.630
	Job type (2)	**1.516**	0.594	6.507	4.553	1.421	14.594
	Job type (3)	−0.054	0.714	0.006	0.948	0.234	3.843
	Job type (4)	0.558	0.625	0.797	1.747	0.513	5.948
	Job type (5)	**1.831**	0.491	13.901	6.238	2.383	16.329
	Job type (6)	0.569	0.465	1.496	1.766	0.710	4.395
	Mood	0.101	0.074	1.842	1.106	0.956	1.280
	Effects of exercise	**−0.782**	0.178	19.265	0.457	0.322	0.649
	Expectations of exercise	**0.254**	−0.782	11.402	1.289	1.112	1.493
	Belief of exercise	**0.104**	0.254	7.375	1.110	1.029	1.196
	Intercept	−0.576	0.104	2.487	0.562		
Screened data							
Final model	Institution (1)	**−0.891**	0.306	8.505	0.410	0.225	0.747
	Sleep	0.148	0.098	2.270	1.159	0.956	1.405
	Effects of exercise	**−0.553**	0.162	11.599	0.575	0.418	0.791
	Expectations of exercise	0.155	0.063	6.142	1.168	1.033	1.320
	Belief of exercise	**0.108**	0.041	6.820	1.114	1.027	1.209
	Intercept	0.216	0.232	0.861	1.241		

**Bolded** parameter estimates or Wald statistics indicated significance at 0.05 level and *italicized* numbers at 0.10 level.

For the *screened data*, compared with the university professionals, the hospital professionals had 0.410 times the odds of selecting this challenge (CI: 0.225, 0.747). For every unit increase in “Exercise effects,” there was about a 58% decrease in selecting this challenge (CI: 0.418, 0.791). For every unit increase in “Belief in exercise,” there was about an 11% increase in the event (CI: 1.027, 1.209). The R-square was 0.214, and the GOF was 0.577.

## 5. Discussion

This section started with the discussions on which groups were more likely to be flagged as careless respondents. The rest of the discussion evolved around the tests of associations and logistic regressions.

### 5.1. Demographic variables in careless responses

A series of chi-square tests were performed. The chi-square test on the contingency table for gender and deleted group (1 = deleted and 0 = not deleted) produced a significant test statistic (test statistic = 10.878 with *df* = 1, a *p*-value of 0.001). The male was more likely to fill out the survey carelessly. The chi-square test for the institution × deleted group was also significant (test statistic = 7.177 with *df* = 1, a *p*-value of 0.007). Those at universities were more likely to produce CR. Similarly, those with less experience (i.e., less number of years working in the profession) and those with lower levels of education were significantly more likely to behave carelessly (*p*-value <0.000 and *p*-value of 0.003 respectively).

### 5.2. What did we understand from tests of associations?

There were some similarities between both approaches in Table 3. The challenge of “Helping students’ academic growth” was significantly associated with institutions in both approaches. Compared with hospital professionals, more university professionals consider it challenging to help students grow academically. This association was significant for both approaches. The job of university professionals was to help students grow. Their experiences with students’ academic development were substantively more than those at hospitals. For both approaches, gender and years of experience were significantly associated with the challenge of “Peer Cooperation.” For both approaches, marital status was significantly associated with “Professional communication with peers”.

“Challenge of multidisciplinary learning” was not significantly associated with demographic variables in both approaches. Several explanations exist. First, our sample size was not enough to produce a significant relationship. More participants might solve the issue. Second, the relationship between this challenge and demographic variables was suppressed by a mediator. Third, Chinese public health professionals might understand “multidisciplinary” differently. If knowledge, skills, and strategies from multiple disciplines are related to their career or job, they might classify these into their majors instead of understanding them as multidisciplinary.

Differences existed. More tests yielded smaller *p-*values with the screened dataset, as indicated by * signs in Table 3. Those marked by ** signs in Table 3 indicated larger *p*-values for the screened data. Education level was significantly associated with “Improving my job” for the conventional data but insignificant for the screened data. Comparing the results for the two approaches, there were more tests with smaller *p*-values in the screened data. Thus, the significance levels of the uncleaned data were obscured in many cases.

### 5.3. What did we understand from logistic regression?

In the discussion of logistic regression, only the variables at 0.05 levels were presented in 4.31 and 4.3.2.

#### 5.3.1. Which demographic features were more important?

In the current study, years of experience and job type mattered. The professionals in directors or deputy chief positions and those with master’s degrees had significantly higher odds of choosing “Improving my job” as a challenge. For the conventional data, teaching doctoral or master courses and professionals in directors or deputy chief positions were more likely to endorse “Helping students’ academic growth” as a challenge. With the nature of their job, they encountered more student issues. The significance varied with demographic variables.

#### 5.3.2. Which variable had stronger predictive power?

In the current literature, exercise has been conceptualized as uni-dimensional. For example, exercise was some workout each time in the national report (13). In our study, exercise was multi-dimensional. “Expectations of exercise” was a frequent predictor for PD challenges. “Expectations of exercise” impacted the professionals’ realization of their professional challenges. The coefficients of “Exercise Effects” were negative, suggesting more desirable effects health professionals had from exercise, the more likely they were to rate PD challenges as easy. “Belief in exercise” was also predictive.

“Sleep” predicted “Peer cooperation” in both approaches. Cynicism predicted “Improving my job” and “Professional communication with peers” for the screened data. The negative coefficient associated with professional efficacy suggested that those with higher professional efficacy considered this challenge easy. This aligns with existing research on the positive impact of professional efficacy on PD (29–31). “Friends” predicted “Peer cooperation” negatively at 0.05 for the conventional approach. Burnout did not predict PD as much as exercise did.

#### 5.3.3. Correctly classified participants

**Figure 3:**
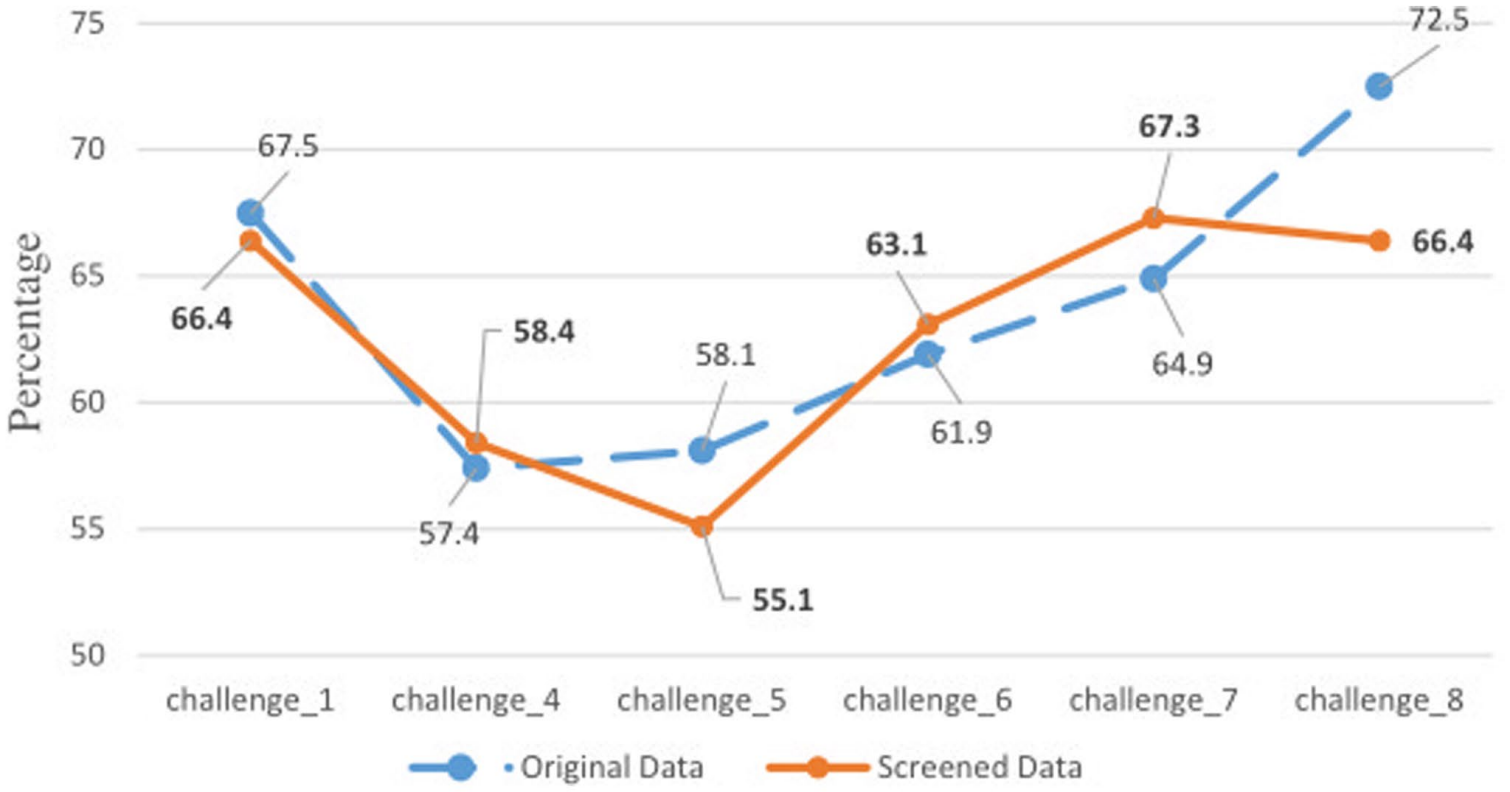
Percentages of correct classification for two approaches. **Bolded** numbers were from the screened data. See the bottom of Table 3 for challenge information.

Figure 3 presents the percentages of correct classification for the selected models of the two approaches. For “Helping students’ academic growth,” the conventional approach outperformed the data screening approach (72.55% versus 66.4%). For “Multidisciplinary learning,” the conventional approach was better than the data screening approach (58.1% versus 55.1%). Apart from these two conditions, the percentages of correct classifications were very close for the two approaches. This confirmed that the removed data were invalid.

#### 5.3.4. Implications

First, exercise can enhance PD. The findings on exercise were consistent with the literature, which supports the positive role of exercise on learning or learning-related motivation (12, 55, 56). Clinically, professionals should develop good exercise habits and proper expectations toward exercise results to facilitate their PD and develop self-efficacy. Those with burnout syndrome should also have adequate exercise to reduce burnout (13). Institutions should have policies to encourage employees to exercise.

Second, the findings on burnout and burnout-related issues suggest that professionals should build a network of supportive friends who can positively influence them to enhance their professional self-efficacy. Individuals and institutions should create an environment to foster PD and professional efficacy (13).

Lastly, the findings in 4.3.3 were consistent with the survey literature, which proves the uselessness of removed CR (e.g., 39–41). Public health researchers should examine the data quality before conducting any research.

## 6. Conclusion

The current research provided an understanding of Chinese public health professionals and their perceived PD challenges. For both approaches, the sub-dimensions of burnout did not significantly predict the challenges frequently as the sub-dimensions of exercise. Some variables did not enter the models. Some mediators might exist, suppressing the relationship between the independent and dependent variables. The correct classification rates with both approaches were close.

Future research may follow several directions. First, the instrument in the current study was of mixed scales. It can be revised such that all items are on the same scale, enabling researchers to check the data quality of all items. Second, the current research only applied two CR detection techniques. Other CR techniques can be used. Third, our sample size was 265 and 214 for the conventional and data screening approaches. It is possible for future research to recruit more participants.

## Data availability statement

The original contributions presented in the study are included in the article/Supplementary material, further inquiries can be directed to the corresponding author.

## Ethics statement

The studies involving humans were approved by Research Office, Shandong Youth University of Political Science, Jinan, Shandong, China. The studies were conducted in accordance with the local legislation and institutional requirements. The participants provided their written informed consent to participate in this study.

## Author contributions

YW participated in every research step (instrument development and revision, data collection and cleaning, data analysis, manuscript writing, and revision). HZ provided insight concerning the research design and participated in the instrument development and validation, data collection, and data cleaning. XK participated in the instrument development and validation, and data collection. FL participated in the instrument development and data collection. YW, XK, FL, and HZ listed meet the authorship criteria according to the latest guidelines of the International Committee of Medical Journal Editors. All authors contributed to the article and approved the submitted version.

## Conflict of interest

The authors declare that the research was conducted in the absence of any commercial or financial relationships that could be construed as a potential conflict of interest.

## Publisher’s note

All claims expressed in this article are solely those of the authors and do not necessarily represent those of their affiliated organizations, or those of the publisher, the editors and the reviewers. Any product that may be evaluated in this article, or claim that may be made by its manufacturer, is not guaranteed or endorsed by the publisher.

## Footnotes

^1^ https://www.apha.org/about-apha/centers-and-programs/center-for-professional-development
